# A community assessment of privacy preserving techniques for human genomes

**DOI:** 10.1186/1472-6947-14-S1-S1

**Published:** 2014-12-08

**Authors:** Xiaoqian Jiang, Yongan Zhao, Xiaofeng Wang, Bradley Malin, Shuang Wang, Lucila Ohno-Machado, Haixu Tang

**Affiliations:** 1 Division of Biomedical Informatics, Department of Medicine, University of California, San Diego, La Jolla, CA 92093, USA; 2 School of Informatics and Computing, Indiana University, 150 Woodlawn Avenue, IN 47401 Bloomington, USA; 3 Department of Biomedical Informatics, School of Medicine, Vanderbilt University, Nashville, TN 37203 USA

**Keywords:** Genome-wide association studies, Data sharing, Privacy protection, Differential privacy

## Abstract

To answer the need for the rigorous protection of biomedical data, we organized the Critical Assessment of Data Privacy and Protection initiative as a community effort to evaluate privacy-preserving dissemination techniques for biomedical data. We focused on the challenge of sharing aggregate human genomic data (e.g., allele frequencies) in a way that preserves the privacy of the data donors, without undermining the utility of genome-wide association studies (GWAS) or impeding their dissemination. Specifically, we designed two problems for disseminating the raw data and the analysis outcome, respectively, based on publicly available data from HapMap and from the Personal Genome Project. A total of six teams participated in the challenges. The final results were presented at a workshop of the iDASH (integrating Data for Analysis, 'anonymization,' and SHaring) National Center for Biomedical Computing. We report the results of the challenge and our findings about the current genome privacy protection techniques.

## Introduction

The biomedical community is evolving to benefit from and to contribute to big data science. This began with advances in high throughput and computing technologies and is expanding to include expected, as well as unexpected, sources of health-related data, such as electronic health records and social media. A large amount of biomedical data (e.g., human DNA sequences) are being rapidly generated in research and clinical laboratories and healthcare settings [1]. Human genome sequencing will soon become a routine procedure in biomedical research and healthcare, thanks to the rapidly increasing throughput and decreasing cost of DNA sequencing techniques.

To transform such data into knowledge that is applicable to biomedicine, new technologies for large-scale investigations and meta-analysis analysis (e.g., [2]) on genomic data continue to be developed, increasing the probability that human genomes will be used for clinical diagnosis and therapy, a trend dubbed "base pairs to bedside" [3].

However, further progress in this area has been impeded by the constraints in accessing human genome data, due in part to privacy concerns related to the disclosure of these data [4,5]. As human genome is a type of biometric identifier, special provisions to protect privacy of individuals need to be taken into account.

It is well known that sharing raw DNA data (e.g., genotypes) poses risks even after the removal of explicit identifiers (e.g., name and Social Security number.), as the donor can be possibly re-identified through the genetic markers (e.g., single nucleotide polymorphisms, or SNPs) in the data [6-8]. Even aggregated DNA data, such as allele frequencies, have been found to leak out identity information [9]. Specifically, Homer et al. [10] discovered that the presence of an individual in a case group can be reliably determined from allele frequencies using the person's DNA profile. As a result, NIH and Wellcome Trust removed most aggregated DNA data from the public domain to protect the privacy of study participants [11]. Computational approaches have been developed to characterize which SNPs could be shared under the assumption that they are common variants [12,13]. For example, it was shown that re-identification is unlikely if the set contains more than 1000 people [13]. Nonetheless, data owners are becoming more cautious about sharing human genomic data, often implementing additional processes to review proposals for data access.

In the past several years, there is growing interest in developing effective methodologies to analyze and disseminate sensitive data. Cryptographic protocols (such as homomorphic computation [14-17]) were developed for analyzing sensitive data through computation over the ciphertext. However, these methods provide no protection to the output of computation, and thus cannot be directly used for disseminating sensitive data. Data perturbation techniques (e.g., [18-20]) achieve privacy protection on input and/or output data and can be used to disseminate sensitive data or to publish computing results that contain sensitive information. A recent review on privacy preserving technology to support data sharing for comparative effectiveness research presents an overview of some of these techniques [21].

A critical challenge of applying privacy technology on human genomic data is to balance data sharing (and data utility in real-world genomic analyses in particular) and privacy protection. To investigate the extent to which data perturbation technologies are practical, we organized the Critical Assessment of Data Privacy and Protection (CADPP) Workshop as a community effort to evaluate the effectiveness of these methodologies (in particular differential privacy approaches [20]) for genomic data. This workshop was organized around two specific problems. For the first problem, teams focused on the challenge of sharing aggregate human genomic data (e.g., allele frequencies) to preserve the privacy of the data donors, without undermining the utility of the data in genome-wide association studies (GWAS). This means that, in practice, the most significant genomic regions identified by a GWAS were preserved after data perturbation. In the second problem, teams were challenged to publish GWAS results (i.e., the most significant genomic regions) that meet the differentially privacy criteria under a specific privacy budget.

We devised a task for each of these two problems, based on publicly available data from the International HapMap Project [22] and the Personal Genome Project (PGP) [23]. A total of six teams from two countries participated in the challenges and submitted their results to one or both tasks. In this article, we describe the design of the challenge tasks and our findings. Two companion papers in this issue of the journal [22,23] written by participating teams provide details of the data perturbation techniques that were used in the challenge tasks.

## Background

For both tasks, we challenged teams to apply data perturbation techniques based on the differential privacy standard. These techniques attempt to add noise to the allele frequencies of the *case group *so that the resulting data are indistinguishable when any individual is present or absent in the case group. Below, we provide background on differential privacy, a framework developed by Dwork [20], for readers who are not familiar with this model.

**Definition 1 ***(* ε*-Differential Privacy) A randomized algorithm * f*satisfies * ε*-differential privacy if for all adjacent datasets * D*and *$D^{\prime }$*, and any possible output *$\hat{D}$*in the output space of * f,

(1) $$\frac{Pr\left[f\left(D\right)=\hat{D}\right]}{Pr\left[f\left(D\text{'}\right)=\hat{D}\right]}\leq e^{\varepsilon }$$

Note that $\hat{D}$ can be a dataset or a numerical value (a differentially private query result such as a statistic) depending on the application of interest. The Laplacian mechanism [24] is commonly applied in data perturbation methods to achieve differential privacy. It adds noise drawn from a Laplacian distribution, Laplace(0,Δf/ε), to the output of a computation on the dataset. The degree of noise depends on the sensitivity of the computation. In this model, sensitivity characterizes the maximum change in the output of a function when elements change in a dataset.

**Definition 2 ***For any *$f:D\to R^{d}$*, and all adjacent datasets * D* and *$D^{\prime }$*, the sensitivity of * f*is*

(2) $$\text{Δ}f=\text{ma}{\text{x}}_{D,D^{\prime }}||f\left(D\right)-f\left(D^{\prime }\right)|{|}_{1}$$

Another popular method for achieving differential privacy is called the exponential mechanism, which was proposed by McSherry and Talwar [25]. This mechanism chooses an output tεT that is close to the optimum with respect to a utility function while preserving the differential privacy definition. The exponential mechanism takes as input a data set  D, an output range  T, a privacy parameter  ε, and a utility function $u:\left(D\times T\right)\to R$ that assigns a real-valued score to every possible output tεT, for which a higher score stands for better utility. The mechanism induces a probability distribution over the range of εT and sample an output  T in proportion to $\text{exp}\left(\frac{\in u\left(D,t\right)}{2\text{Δ}u}\right)$, where $\text{Δ}u=\text{ma}{\text{x}}_{D,D^{\prime }}||u\left(D\right)-u\left(D^{\prime }\right)|{|}_{1}$ stands for the sensitivity of the utility function.

## Method

As mentioned, we organized the challenge around two specific problems: i) sharing allele frequencies and ii) sharing the most significant single nucleotide variants (SNVs) in GWAS.

We devised one task for each problem. For each task, we constructed two datasets based on case and control groups of individuals. The case group was composed of the subjects from the Personal Genome Project (http://www.personalgenomes.org/), which consisted of 411 participants whose genotypes were profiled on 29,757,319 SNVs across the whole genome. The control data consisted of 174 participants from the CEU population in HapMap (http://hapmap.ncbi.nlm.nih.gov/index.html.en).

### Task 1: privacy preserving data sharing

The goal of this task is to achieve the highest utility under the perturbed case-control test statistics (i.e., allele frequencies). Each team was provided with the original allele frequencies for the case group, and was instructed to submit perturbed allele frequencies for this group. Note that, in the challenge, we assume the genotypes of the control group are publicly shared, and thus there is no need to protect them. In order for participating teams to evaluate the effectiveness of their data perturbation method, the control group data were made available.

#### Quantification of privacy risks

Numerous statistical attack models have been proposed to detect the presence of a participant in a case population. Homer et al. presented a statistical inference approach that computes the likelihood of a patient to participate in a case group whose aggregate allele frequencies on many SNV were shared [10]. Following this approach, Sankararaman et al. [13] showed the upper bound of the power by using the strongest re-identification test statistic, i.e., the likelihood ratio (LR) test, which can be written as

(3) $$\bar{L}=\overset{m}{\underset{j=1}{\sum }}(x_{j}log\frac{{\hat{p}}_{j}}{p_{j}}+\left(1-x_{j}\right)log\frac{1-{\hat{p}}_{j}}{1-p_{j}}),$$

where $x_{j}$ is the allele type (i.e., 0 for the major allele, or 1 for the minor allele) at the SNV site *j, m *is the total number of SNVs, $p_{j}$ is the minor allele frequency of SNV *j *in the case group, and ${\hat{p}}_{j}$ is the corresponding number for the reference group.

A statistic ${\bar{L}}_{i}$ is then computed over all SNV sites for a subject *i*, representing the likelihood of that subject's presence in the case group. The probability that a subject in the case group will be re-identified is considered to be *high *if the LR test statistic from her SNVs is significantly greater than those of subjects who are not in the case group.

We used the LR test to evaluate the privacy risks in the submitted data after perturbation from participating teams. The submitted data were considered sufficiently protected if there were approximately the same number of individuals in the case group with LR statistics above the same threshold as those in a test group (i.e., individuals not in the case or control groups). The utility of the perturbed data were measured using the number of significant SNPs identified by the *χ*^2^-association test [26], which is commonly used in GWAS for case and control groups. A data perturbation method was considered to have high utility if a majority of the significant (below a *p*-value threshold, e.g., 10^−5^) SNVs reported by the *χ*^2^-association test on perturbed data were *true*. In other words, these SNVs were identified as truly significant on the original data using the same statistical test and a majority of truly significant SNVs could be identified on the perturbed data.

We implemented the likelihood ratio test as an online tool (http://www.humangenomeprivacy.org/service.php) for participating teams to evaluate the privacy risks in their perturbed data. A team could upload perturbed allele frequencies in the case group, and the LR statistics were calculated for each case individual using his/her actual genotype, the uploaded allele frequencies in the case group, and the actual allele frequencies in the control group (from the HapMap project). For comparison, the LR statistics were also computed for a group of test individuals (also from the HapMap project) who were not in either the case or control group. If the number of case individuals was not significantly larger than the number of test individuals who had a test statistic above a certain threshold, these case individuals were considered indistinguishable from any individuals outside the case group and the perturbed case group data were considered to be privacy-preserving.

We released two datasets for this task. The first dataset consisted of 311 SNV sites spanning of 5M bps genomic segment on human chromosome 2, which was a challenging dataset for which the re-identification risk could be alleviated (but not fully mitigated) using our baseline method (see below). The second dataset consisted of 600 SNV sites spanning a 1M bps genomic segment on human chromosome 10, which is a moderately challenging dataset whose re-identification risk can be fully mitigated by our baseline method.

#### Baseline method

To ensure the feasibility of the competition, we implemented two baseline algorithms for adding noise to data used in task 1, which we refer to as the *naïve *and *advanced *algorithms. In the naïve algorithm, we treated the allele counts across multiple SNP sites as a histogram, and added Laplacian noise to the allele counts. Note that the sensitivity of the count function over *N *SNVs is 2*N*, which can be used inLaplace(0,Δf/ε) to determine the amount of noise that needs to be added.

As the name implies, the naïve algorithm is simple and straightforward. However, the sensitivity in this case is high because the dimensionality of the dataset, which corresponds to the number of SNVs, is large (e.g., several hundred in the case of the two datasets used in challenge 1). The high sensitivity can resulting in adding a large amount of noise to the data, which may significantly damage the utility.

By contrast, the advanced data perturbation algorithm is designed based on the *haplotypes *[27], an intrinsic feature of the human genome. Each copy of the DNA sequence in a single human genome can be partitioned into *haplotype blocks *(or *haploblocks*); within each block, a specific combination of alleles across multiple adjacent SNV sites is called a haplotype. Due to linkage disequilibrium (LD) in human, SNVs within each block are likely inherited together. As a result, inter-haploblock SNVs are more highly correlated than intra-haploblock SNVs. Therefore, the number of potential SNV sequences in each haplotype block (across *n *SNV sites) is much smaller than the theoretical bound (i.e., $2^{n}$). Notably, the haplotype block structure of the human genome can be inferred from public data, which are independent from the sensitive case data to be protected [27].

**Figure 1:**
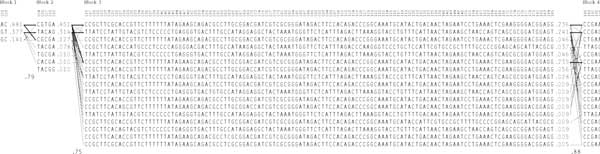
**The haploblock structure in the first dataset used in task 1**.

Figure 1 shows the first three haploblocks of dataset 1 that were used in task 1. The SNV sites and the haplotypes are shown below the haploblock ID. The numbers next to each haplotype represent their observed frequencies in the CEU population of the HapMap project. The solid lines between haplotypes from adjacent haploblocks represent the most common combinations between the haplotypes. The thickness of the line represents the frequencies of the corresponding combinations. From the figure, it can be seen that about 50% of all SNV sequences contain the combination of haplotypes ACCGTGA in the first two haplotype blocks, and the third haplotype block consists of 125 SNVs, but has only 17 common haplotypes (instead of the theoretical upper bound of 2^125^). Rather than adding noise to allele counts on individual SNV sites, the advanced data perturbation approach adds Laplacian noise to the haplotype counts, which are normalized within each haplotype block.

We derived the allele counts on each SNV site from counts of haplotypes containing the site. By using haplotypes, we can reduce the dimensionality of genomic data by one order of magnitude. In practice, we need to allocate the privacy budget (default 1) into haploblocks in an unequal manner to ensure it is not overspent. To do so, we employed an empirical method for budget allocation. Specifically, we allocated a budget to each haploblock proportionally to the number of haplotypes within that block. In doing so, a larger budget will be assigned to the blocks with a greater number of haplotypes (and thus less noise will be added to them). Similarly, a smaller budget will be assigned to the blocks with fewer haplotypes. Note that the total privacy budget across the entire dataset remains the same as in the naïve approach.

#### Challenge datasets and utility evaluation

**Figure 2:**
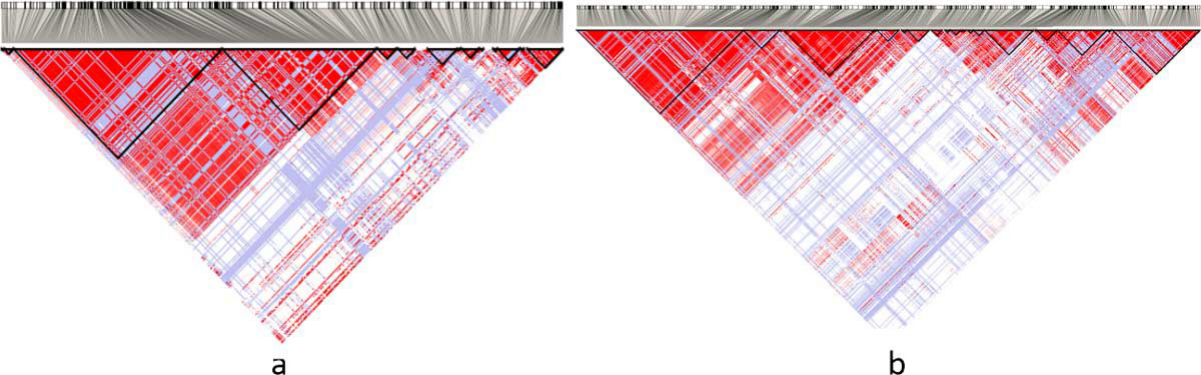
**The haploblock structure in the datasets used in task 1**. (a) The haploblock structure for dataset 1. (b) The haploblock structure for dataset 2.

We constructed two datasets for Challenge 1. The first case dataset consists of 311 SNVs from 200 PGP participants. As shown in Figure 2(a), the SNVs span 14 consecutive haplotype blocks in one genomic locus from 29504091 to 30044866 on human chromosome 2. The control and test datasets comprise the corresponding SNVs from 174 HapMap CEU individuals, which are publicly available.

**Figure 3:**
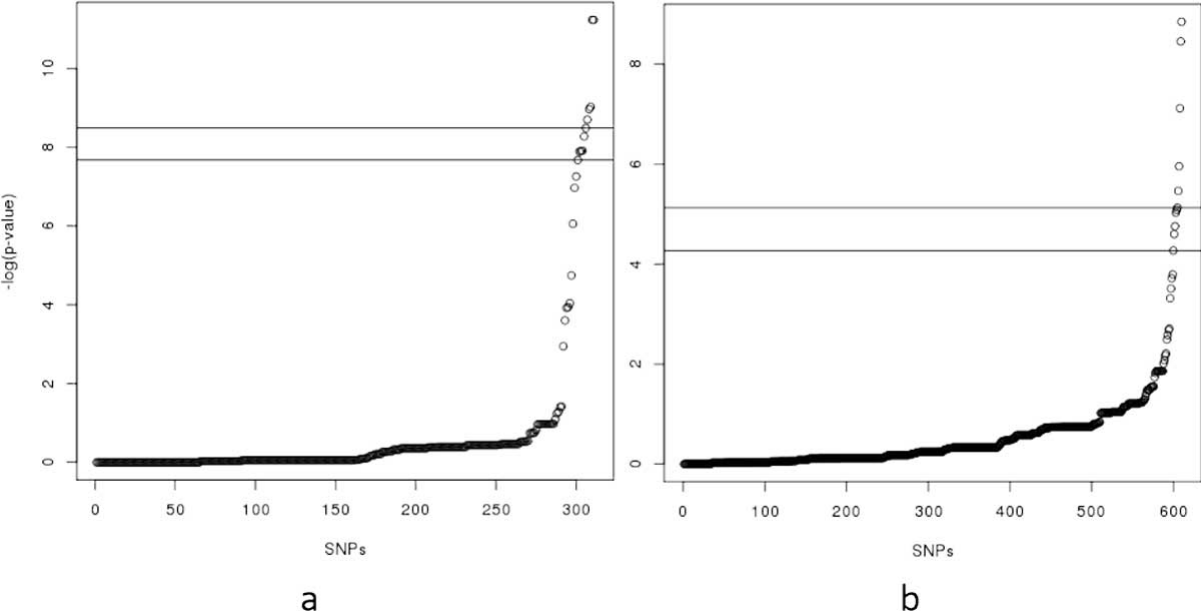
**The p-value distributions based on a *χ*^2 ^test (a) The *p*-value distribution for dataset 1**. (b) The *p*-value distribution for dataset 2.

Figure 3(a) shows the *p*-value distribution, based on a *χ*^2 ^test, of all SNVs in this this case-control dataset. The upper and lower lines in the figure differentiate the top-5 and top-10 most significant SNVs, respectively, from the rest of the set, which correspond to the *p*-value cutoffs of 10^−8 ^and 10^−7^, respectively. The practical p-value threshold used in GWAS is 10^−5^, indicating that there exist many significant SNVs in the dataset to be identified in typical GWAS analyses.

**Figure 4:**
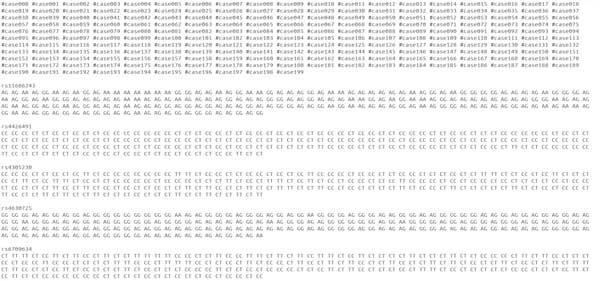
**A snapshot of the dataset released for task 1**.

The second case dataset consists of 610 SNVs of the same 200 participants from the PGP as the first dataset. As shown in Figure 2(b), these SNVs span over 21 consecutive haplotype blocks, which are in one genomic locus from 55127312 to 56292137 on human chromosome 10. The control dataset is comprised of the corresponding SNVs from the 174 HapMap CEU individuals. Figure 3(b) shows the different levels of significance according to the *χ*^2^-association test on this dataset. Figure 4 shows the file format of the data for task 1 that were provided to participating teams (available at http://www.humangenomeprivacy.org/competitiondata.php). The first line of the file consists of the IDs of human individuals, followed by the SNV ID and the genotypes in each individual, which themselves follow the order of SNV site locations. The allele frequencies for the SNVs can be derived from the input data. To evaluate the utility of the perturbed case data submitted by participating teams, we conducted a *χ*^2 ^test on a combination of the submitted and unperturbed control data. The significant SNVs (i.e., *p*-value below a threshold of 10^−5^) were compared to the significant SNVs reported by the same test on the un-perturbed case data. A larger number of common SNVs between these sets indicates higher utility of the corresponding perturbation technique and vice versa.

### Task 2: secure release of analysis results

The goal of the second task was to evaluate the utility of competing data perturbation techniques in releasing privacy protected results of GWAS. We consider a typical GWAS, where the users are interested in knowing the identity of the top-*K *(e.g., *K *= 1, 5 or 10) most significant SNVs among all SNVs (for the statistical test) across the genome. Again, we used the *χ*^2 ^test statistic for utility evaluation in the case-control analysis. We expected the released GWAS results to achieve a given privacy standard: they should be *differentially private *with a privacy budget of 1. The utility of the anonymized GWAS results submitted by the participating groups was evaluated based on the proportion of SNVs in the released result that were among actual top-*K *most significant SNVs that would have been released if preserving privacy was not a concern.

We first preprocessed the case data containing 29,755,199 SNV sites to make them compatible with the control data. Many SNV sites were genotyped for only a small portion of PGP individuals, and some other SNV sites genotyped in PGP were not genotyped in HapMap. We removed all unmatched SNV data between these two datasets and retained only the *valid *2,500,781 SNV sites genotyped in at least 174 PGP participants so that we could estimate the allele frequencies on these sites accurately. Next, we eliminated PGP participants from our constructed dataset who had fewer than 90,000 genotyped valid SNV sites. After these preprocessing steps, there were still genotypes missing for some PGP participants, which were imputed based on allele frequencies from the CEU population in HapMap using fastPHASE, a commonly utilized phasing tool [28]. Finally, we constructed the case group consisting of 201 PGP participants, and a control group consisting of 174 HapMap CEU participants, both genotyped on the same set of 106,129 SNV sites.

**Figure 5:**
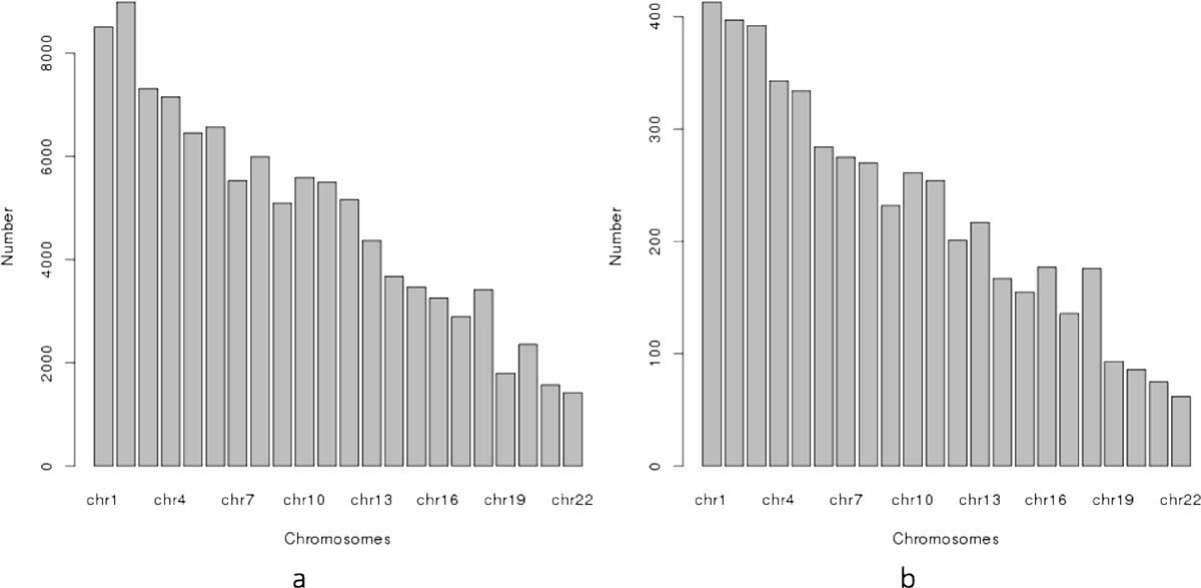
**The number of SNVs sampled from chromosomes**. (a) The number-sampling distribution for the large dataset. (b) The number-sampling distribution for the small dataset.

We constructed two datasets for this task. The large dataset consists of all valid 106,129 SNVs from 201 cases and 174 controls after the data pre-processing procedure, which was used to evaluate the utility of the perturbation algorithms submitted by participating teams. We also constructed a small dataset (a subset of the larger one) consisting of 5000 SNVs on the same case and control groups that were randomly sampled from the larger dataset. The SNVs in both datasets were evenly distributed in different human chromosomes (Figure 5), indicating that this represented a good training set for participating teams to test their algorithms.

### WIDGET: a web interface for dynamic genome-privacy evaluation

We built a companion webservice using JavaScript and R Shiny server technology (http://shiny.rstudio.com/) to dynamically compare the algorithms of participating teams that can be accessed at https://humangenomeprivacy.ucsd-dbmi.org. Using this webservice, a user can assess competing models in finer granularity than what is reported in this article. The figures below illustrate how WIDGET can be used to evaluate a model's performance for task 1 and task 2, respectively.

**Figure 6:**
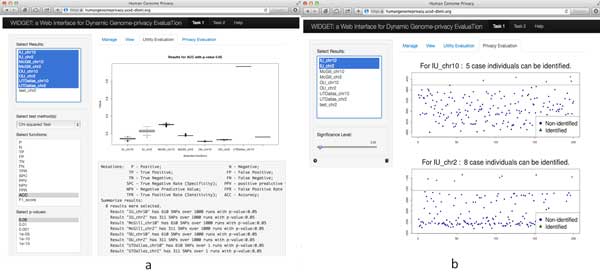
**Evaluation results for task 1: (a) data utility and (b) privacy evaluation through WIDGET**.

Figure 6(a) shows boxplots indicative of the accuracy of different privacy-preserving synthetic data generation algorithms (Task 1). In addition to accuracy, users can check other metrics (e.g., true positive rate, false positive rate, and F1), which are listed under the boxplot. In Figure 6(b), we illustrate the privacy risk using the LR test based on a given significant level. Users can compare multiple results at a time, where identified and non-identified individuals are illustrated with different markers. Users can adjust the significance level in their studies and any change is dynamically updated in the boxplots.

**Figure 7:**
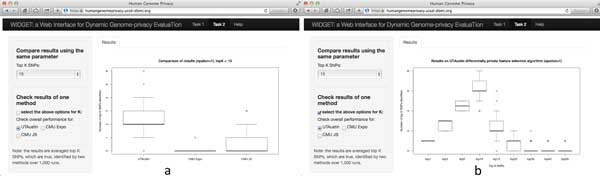
**Evaluation results for task 2: (a) comparison of different methods for a given K in the top-K SNV identification challenge; (b) comparison of different top-K SNV identification performance of a given method**.

In Figure 7, we show how to use WIDGET to compare three methods and *K *values (up to 9) for top-*K *SNV identification (Task 2), where the same privacy standard (i.e., ∈=1) was used for all cases. Figure 7(a) depicts the number of SNVs identified by three methods under *K *= 15. Figure 7(b) illustrates the top-*K *(e.g., 1, 3, 5, ..., 50) SNV identification performance of a given method.

## Results and discussions

A total of six teams participated in the competition. Three teams, from the University of Oklahoma, University of Texas (UT) Dallas, and McGill University, participated in the first task. The team from Indiana University collaborated with the UCSD organizers to design and implement the baseline methods, but they did not participate in the competition. Two teams, from UT Austin and Carnegie Mellon University, participated in the second task. The results of the challenge were presented at an iDASH (a National Center for Biomedical Computing based at UCSD) workshop on March 24, 2014 in La Jolla, California. All teams presented their methods and results at the workshop. The details of the methods used in the challenge are described in separate articles in this issue [29,30]. Below, we summarize the results of the challenge and our own findings.

Table 1 shows the results of the evaluation methods for task 1. The privacy risk in the perturbed data was measured using the *power *of the LR test; i.e., the fraction of case individuals who could be re-identified with confidence higher than a specified threshold. If the power was not significantly higher than 0.05, we considered the perturbed data to have a small privacy risk. From the results, we can see that in all cases except two (including the haplotype-based method on the second dataset), the privacy risk was sufficiently mitigated by the data perturbation techniques. We then measured the utility of the perturbed data based on the number of significant SNVs detected by using *χ*^2 ^test (see above) under various *p*-value cutoffs (0.05, 0.001, and 0.00001) of a *χ*^2^test.

**Table 1:** Results of Task 1.

		Baseline		Team 1	Team 2	Team 3	# of sig SNVs
		**SNV-based**	**Haplotype-based**	**U Oklahoma**	**UT Dallas**	**McGill U**	

D1	Power Cutoff 5.00E-02 1.00E-03 1.00E-05	0.05 TPR. FPR 0.864. 0.844 0.632. 0.774 0.643. 0.700	0.03 TPR. FPR 0.910. 0.612 1.000. 0.493 1.000. 0.475	0.61 TPR. FPR 1.000. 0.941 1.000. 0.884 1.000. 0.879	0.04 TPR. FPR 1.000. 0.855 1.000. 0.791 1.000. 0.737	0.01 TPR. FPR 1.000. 0.886 1.000. 0.798 1.000. 0.737	22 19 14

D2	Power Cutoff 5.00E-02 1.00E-03 1.00E-05	0.04 TPR. FPR 0.933. 0.924 0.800. 0.862 0.625. 0.788	0.115 TPR. FPR 0.978. 0.804 1.000. 0.708 1.000. 0.504	0.005 TPR. FPR 1.000. 0.958 1.000. 0.909 1.000. 0.876	0.01 TPR. FPR 0.533. 0 1.000. 0 1.000. 0	0.09 TPR. FPR 0.956. 0.746 1.000. 0.582 1.000. 0.425	45 15 8

In the first column, D1 refers to 200 participants, 311 SNVs (~29504091-30044866, chr2) and D2 refers to 200 participants, 610 SNVs (~55127312-56292137, chr10). The rows labeled 'Power' indicate the ratio of identifiable individuals using the likelihood ratio test in the case group. The other rows start with a cutoff threshold for the *χ*^2^test (e.g., 5 × 10^-^^2^, 10^-^^3^, 10^-^^5^), for which two measurements (true positive rate and false positive rate for SNVs using the *χ*^2 ^test) were calculated under each method. The last column corresponds to the number of significant SNVs calculated using the original data (i.e., without added noise).

The results of the SNV-based algorithm (the naïve baseline method) showed the least utility. It missed a substantial number of truly significant SNVs, while it generated a large number false positive SNVs. The haplotype-based algorithm exhibited better performance. Almost all truly significant SNVs were detected in both datasets, and the number of false positives was reduced. The results of UT Dallas and McGill University teams showed similar results on utility of perturbed data, and nearly all significant SNVs were detected, although both had a high false positive rate. Overall, the McGill University team performed the best in the task and won the task 1 challenge.

Table 2 summarizes the findings from task 2. Notably, both teams achieved impressive performance levels. For the small dataset, when *K *is set to be smaller than 15, both algorithms can correctly identify more than 80% of the top-*K *most significant SNVs. The performance is, however, inversely correlated with *K*, such that when *K *≥ 15, both methods return only around 50% of the true top-*K *SNVs. Similarly, for the large dataset, when *K *< 10, both algorithms perform well.

**Table 2:** Results of Task 2.

	Teams	Top 1	Top 3	Top 5	Top 10	Top 15	Top 20	Top 30
Small (5000 SNVs)	UT Austin CMU	1 0.98	2.66 2.28	4.44 3.53	8.48 7.89	7.07 4.59	4.68 2.32	2.37 1.16

Large (100K SNVs)	UT Austin CMU	1 0.98	2.65 2.26	4.41 3.56	5.90 3.27	2.26 0.42	0.69 0.15	0.18 0.07

The table shows the average number of (1000 iterations) privacy-preserving SNV identification algorithms developed by the two participating teams. Both algorithms were trained using the small dataset consisting of 5000 SNVs, and then were tested on both small and large datasets, i.e., select top *K *(i.e., *K *= 1, 3, ..., 30) most significant SNVs.

Overall, the UT Austin team generated the best results. One reason is that their Hamming distance based utility function had relatively smaller sensitivity (therefore reduced perturbation noise) when compared to that of the *χ*^2 ^test statistics used by the CMU team. From the results of task 1, we observed that it remains a challenge to share aggregate human genomic data in a privacy-preserving manner while maintaining truthful associations between the SNV data and the case-control groups. Even for a single genomic locus consisting of a few hundreds of SNVs the association was largely broken after data perturbation.

This problem will become more challenging as larger volumes of human genomic data are planned for dissemination. The performance of differential privacy-based data perturbation techniques heavily relies on the dimensionality of the data. Even though we can reduce the number of dimensions by utilizing unique semantics of genomic data, such as haplotype blocks, it is unlikely that current perturbation techniques will scale well for sharing whole human genomic data.

On the other hand, the results in task 2 showed that privacy-preserving techniques work well for sharing analytical results (by using statistics commonly used in GWAS). High utility can be preserved when only a small number (e.g., 5-10) of the most significant SNVs are of interest to the end users. Notably, this task is well aligned with the centralized computing model, in which a computing center hosts genomic data, as well as the service of customized analyses on these data, and will only release the results of these analyses [31].

## Conclusion

It is essential to develop new privacy-preserving techniques that enable the sharing of human genomic data in a way that preserves the privacy of the data donors, without undermining the utility of the data or impeding its convenient dissemination. This workshop only focused on technical aspects of privacy, but there are many other components, such as social and legal controls, that need to be accounted for when erecting privacy preserving infrastructures. As an initial step to assess existing techniques, we organized the first challenge with two tasks based on real-world human genomic data in GWAS. We discovered that differential privacy-based data perturbation techniques have limitations in sharing a large volume of human genomic data, but can be used to disseminate analytical results (by using GWAS-like statistics), through services like those offered by NCBI. We plan to organize this challenge annually, and hereby welcome suggestions and comments to improve the contest in upcoming years.

## Competing interests

The authors declare that they have no competing interests.

## Authors' contributions

XJ and HT drafted the majority of the manuscript and entire team edited and updated it accordingly. BM and LOM guided the experimental design and provided detailed edits to the manuscript. XW, HT and XJ designed the baseline methods. YZ and SW implemented the evaluation mechanisms for the competition.

## Declarations

Publication of this article has been funded by iDASH (U54HL108460), NHGRI (K99HG008175), NLM (R00LM011392, R21LM012060), CTSA (UL1TR000100), and NCBC-linked grant (R01HG007078)

This article has been published as part of *BMC Medical Informatics and Decision Making *Volume 14 Supplement 1, 2014: Critical Assessment of Data Privacy and Protection (CADPP). The full contents of the supplement are available online at http://www.biomedcentral.com/bmcmedinformdecismak/supplements/14/S1.
